# Immunohistochemistry profiles of breast ductal carcinoma: factor analysis of digital image analysis data

**DOI:** 10.1186/1746-1596-7-27

**Published:** 2012-03-16

**Authors:** Arvydas Laurinavicius, Aida Laurinaviciene, Valerijus Ostapenko, Darius Dasevicius, Sonata Jarmalaite, Juozas Lazutka

**Affiliations:** 1 National Center of Pathology, affiliate of Vilnius University Hospital Santariskiu Clinics, P.Baublio 5, LT-08406 Vilnius, Lithuania; 2 Faculty of Medicine, Vilnius University, M.K.Ciurlionio 21, LT-03101 Vilnius, Lithuania; 3 Institute of Oncology, Vilnius University, Santariskiu 1, LT-08660 Vilnius, Lithuania; 4 Faculty of Natural Sciences, Vilnius University, M.K.Ciurlionio 21, LT-03101 Vilnius, Lithuania

**Keywords:** Immunohistochemistry, Digital pathology, Breast cancer, Androgen receptors, Estrogen receptors, Progesteron receptors, Hypoxia-inducible factor 1α, Special AT-rich sequence-binding protein 1

## Abstract

**Background:**

Molecular studies of breast cancer revealed biological heterogeneity of the disease and opened new perspectives for personalized therapy. While multiple gene expression-based systems have been developed, current clinical practice is largely based upon conventional clinical and pathologic criteria. This gap may be filled by development of combined multi-IHC indices to characterize biological and clinical behaviour of the tumours. Digital image analysis (DA) with multivariate statistics of the data opens new opportunities in this field.

**Methods:**

Tissue microarrays of 109 patients with breast ductal carcinoma were stained for a set of 10 IHC markers (ER, PR, HER2, Ki67, AR, BCL2, HIF-1α, SATB1, p53, and p16). Aperio imaging platform with the Genie, Nuclear and Membrane algorithms were used for the DA. Factor analysis of the DA data was performed in the whole group and hormone receptor (HR) positive subgroup of the patients (n = 85).

**Results:**

Major factor potentially reflecting aggressive disease behaviour (i-Grade) was extracted, characterized by opposite loadings of ER/PR/AR/BCL2 and Ki67/HIF-1α. The i-Grade factor scores revealed bimodal distribution and were strongly associated with higher Nottingham histological grade (G) and more aggressive intrinsic subtypes. In HR-positive tumours, the aggressiveness of the tumour was best defined by positive Ki67 and negative ER loadings. High Ki67/ER factor scores were strongly associated with the higher G and Luminal B types, but also were detected in a set of G1 and Luminal A cases, potentially indicating high risk patients in these categories. Inverse relation between HER2 and PR expression was found in the HR-positive tumours pointing at differential information conveyed by the ER and PR expression. SATB1 along with HIF-1α reflected the second major factor of variation in our patients; in the HR-positive group they were inversely associated with the HR and BCL2 expression and represented the major factor of variation. Finally, we confirmed high expression levels of p16 in Triple-negative tumours.

**Conclusion:**

Factor analysis of multiple IHC biomarkers measured by automated DA is an efficient exploratory tool clarifying complex interdependencies in the breast ductal carcinoma IHC profiles and informative value of single IHC markers. Integrated IHC indices may provide additional risk stratifications for the currently used grading systems and prove to be useful in clinical outcome studies.

**Virtual Slides:**

The virtual slide(s) for this article can be found here: http://www.diagnosticpathology.diagnomx.eu/vs/1512077125668949

## Introduction

The last decade was marked by intense molecular studies of breast cancer recognizing significant biological heterogeneity of the disease and leading to definition of the molecular types. This has opened new perspectives for personalized therapy and development of multiple gene expression-based systems to prognosticate the disease outcomes and assist in therapeutic decisions [1,2]. Despite proven clinical utility of the systems, at least in the context of some categories of breast cancer, they remain relatively expensive, centralized and frequently require fresh frozen tumour specimens.

Due to the limitations of the molecular systems, current clinical practice of breast cancer therapy is largely based upon conventional clinical and pathologic criteria, including mainly tumour stage (T), lymph node involvement (N), histological grade (G), expression of hormone receptors (HR), and hyper-expression and amplification of human epidermal growth factor receptor 2 (HER2) in the tumour tissue [2,3]. The gap between the accumulated knowledge on multiple molecular profiles of the breast cancer and common clinical practice remains open and in some way is compensated by intrinsic biological subtypes adopted by St Gallen in 2011 [4]. The subtypes may be approximated using clinicopathological rather than gene expression array criteria. Therapy recommendations follow the subtype classification: Luminal A disease generally requires only endocrine therapy, which also forms part of the treatment of the Luminal B subtype. Chemotherapy is considered for most patients with Luminal B, HER2 positive, and Triple-negative (ductal) disease, with the addition of trastuzumab in HER2 positive disease [4]. Distinction between the Luminal A and Luminal B subtypes is based on the estimate of proliferative activity of the tumour, measured by the percentage of Ki67-positive tumour cells [4,5] by immunohistochemistry (IHC).

Although the proposed approach provides a bridge between the molecular types of the disease and clinical practice, it is still largely based on semi-quantitative evaluation of estrogen receptor (ER), progesteron receptor (PR), HER2, and Ki67 expression visualized by IHC. The latter method is confined to an issue of defining and then following cut-off values which leads to misclassification of some patients, at least in borderline cases. According to the currently accepted standards, the reproducibility of the IHC tests is suboptimal, the concordance between the methods and laboratories is below expectations for good clinical practice [1]. The improvement in this area could come from standardizing all phases of the IHC (and HER2 FISH) tests [6,7] along with application of image analysis tools to obtain more accurate, reproducible and quantitative results [8,9]. In addition, digital image analysis (DA) providing continuous data of the IHC biomarker expression is an important prerequisite to apply more powerful mathematical analysis tools for tissue-based biomarker research.

In the view of urgent need to improve prognostic classifiers in breast cancer, efforts are being made to use a combinatorial approach revealing new aspects of the disease and promising more reliable stratification of the risk based on combined biomarkers rather than single ones [10]. In essence, it corresponds to the multivariate analysis approach used to develop multiple gene expression-based systems. It has been shown that similar information can be obtained by a combination of relevant IHC markers [11-14], including the heterogeneity of the disease revealed by cluster analysis [15]. However, combined IHC biomarkers proposed up-to-the-date are mostly based on a combination of several biomarkers evaluated qualitatively or semi-quantitatively. Although clinical utility can be achieved already, it is important to employ multivariate analysis methods to exploit broad dynamic range of the IHC DA data. An important exploratory step of the investigation is delivered by factor analysis, revealing independent factors of variation in the data set of multiple IHC biomarkers. Multidimensional data space reduction and extraction of latent variability factors may uncover true biological meaning and informative value of single biomarkers and provide integrated factor scores as quantitative estimates of the biological processes [16]. This may be the only right approach since one biomarker can reflect several biological processes and have different roles in different disease entities. Furthermore, most robust prognostic factors are likely to come in a form of integrated metamarkers derived by multivariate analysis of multimodal data of various aspects (clinical, pathology, molecular, imaging, etc.) of the disease [13].

In our study, we performed an automated image analysis on a set of 10 IHC markers, including the conventional ER, PR, HER2, and Ki67 along with less investigated androgen receptor (AR), BCL2, HIF-1α, SATB1, p53 and p16 on tissue microarrays (TMAs) of 109 patients with ductal carcinoma of the breast. We present the potential of factor analysis of the IHC marker expression data set to reveal biologically and clinically meaningful interdependencies of the breast cancer immunophenotype.

## Materials and methods

### Study population and clinical methods

Tumour samples were prospectively collected from 203 patients with an invasive ductal carcinoma of the breast treated at the Oncology Institute of Vilnius University and investigated at the National Center of Pathology during the period of 2007 to 2009. Informed consent was obtained and documented in writing before study entry. The study was approved by the Lithuanian Bioethics Committee.

The TMAs were constructed from 10% buffered formalin-fixed paraffin-embedded tissue blocks. One millimetre-diameter cores were punched from tumour areas randomly selected by pathologist (4 cores per patient), thus producing 11 TMAs constructed using the tissue arraying instrument (3DHISTECH, TMA Master, Budapest, Hungary). Paraffin sections of the TMAs were cut for IHC (3 μm-thick) and HER2 FISH testing (4 μm-thick).

IHC was performed on the TMA sections using Ultraview DAB detection kit on Ventana BenchMark XT staining system (Ventana Medical Systems, Tucson, Arizona, USA) [17]. Immunohistochemistry for ER, PR, HER2, AR, Ki67, p53, p16, BCL2, SATB1 and HIF-1α was performed using the SP1, 1E2 and 4B5 (Ventana), SP107 (Spring), MIB-1 (DAKO), DO-7 (Novocastra), E6H4 (CINtec), 124 (DAKO), EPR3895 and EP1215Y (Epitomics) antibodies, respectively. The protocols for HER2 fluorescence in situ hybridization analysis were used as described previously [17].

Digital images were captured using the Aperio ScanScope XT Slide Scanner (Aperio Technologies, Vista, CA, USA) under 20× objective magnification. All TMA spots were evaluated on the monitor visually by the pathologist (DD), providing semi-quantitative estimates of the percentage of positive cells and excluding the spots containing inadequate tumour sample or DCIS from further analyses. The estimates were used for quality assurance of DA results and classification into intrinsic subtypes (see below).

Based on the visual pathologist's evaluation of the ER, PR, HER2 and Ki67 IHC results and HER2 FISH results [17], the cases were classified into the intrinsic subtypes: Luminal A (ER and/or PR positive, HER2 negative, Ki67 < 14%), Luminal B, HER2 negative (ER and/or PR positive, HER2 negative, Ki67 ≥ 14%), Luminal B, HER2 positive (ER and/or PR positive, any Ki67, HER2 over-expressed or amplified), HER2 positive, non-luminal (HER2 over-expressed or amplified, ER and PR absent), Triple-negative (ER and PR absent, HER2 negative) [4].

### Digital image analysis

**Figure 1:**
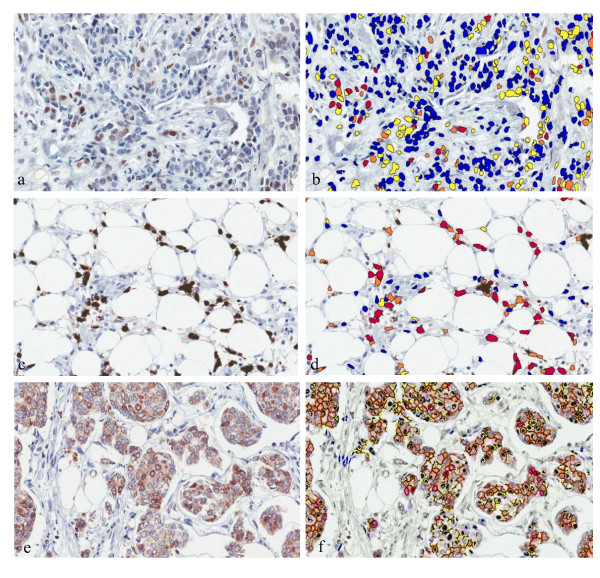
**The examples of immunohistochemistry and digital analysis output images**. Immunohistochemistry and corresponding digital analysis outputs of SATB1 (**a **and **b**), HIF-1α (**c **and **d**), and BCL2 (**e **and **f**). The Nuclear algorithm (b and d) marks the positive cells with color mask according staining intensity (0 - blue, 1+ - yellow, 2+ - orange, 3+ - red). The Membrane algorithm (f) marks the positive cells with complete membranous staining with red outline.

The DA was performed on the same images as the visual evaluation. Aperio Genie Classifier was trained to recognize tumour tissue, stroma and background (glass). The Genie classifier was then combined with Aperio Membrane v9 and Aperio Nuclear v9 algorithms. Respectively, positive tumour cell was defined at the membrane completeness threshold of 50 or the threshold of weak (1+) or higher nuclear staining. The percentage of tumour cells with complete membranous (HER2 and BCL2) staining and positive nuclear (ER, PR, AR, Ki67, p53, p16, SATB1 and HIF-1α) staining was used for further analyses. The examples of IHC and DA analysis output images are presented in Figure 1. The data from all adequate TMA spots were summarized (positive and total cells in the spots were summed, then the percentage of positive cells calculated) into one estimate per patient with a threshold of total number of tumour cells per patient set at > 500. A total of 109 patients with a complete set of 10 IHC markers remained for multivariate analyses.

### Statistical analysis

Summary statistics and distribution analyses were performed with significance tests based on one-way ANOVA and Bonferroni (Dunn) t Tests for pairwise comparisons. Since distributions of HER2, Ki67, HIF-1α, SATB1, p53, and p16 DA results revealed left asymmetry, logarithm-transformed values were used for parametric statistics. For the sake of readability, the prefix "log" is not used in the text or graphs when referring to these markers.

Factor analysis on a DA data set of 10 IHC markers was performed using factoring method of principal component analysis. Five factors were retained based on the threshold of the smallest eigenvalue of 0.85. General orthomax rotation of the initial factors was performed. Factor analyses were performed in two sets of patients: the whole group of ductal carcinoma (n = 109) and HR-positive ductal carcinoma (n = 85) including Luminal A, B, and B HER2 positive tumours.

Pearson's correlation was performed to test the pairwise linear relationships between the continuous variables as a preparatory step for factor analyses. Chi-square test and Fisher's exact test were used to estimate significant associations in non-parametric statistics. Statistical significance level was set at *p <*0.05. Statistical analysis was performed with SAS 9.2 software.

## Results

### Patient and tumour characteristics

The patients' age distribution, tumour stage (T), lymph node status (N), and histological grade (G), based on the Nottingham Grading System [18], are presented in the Table 1. Since the intrinsic subtypes were subdivided based on the visual evaluation of the IHC images, the DA results on ER, PR, HER2, and Ki67 do not strictly correspond to the conventional cut-off values used for the definition of intrinsic subtypes [4]. Pairwise correlations between the IHC markers are presented in the Table 2.

**Table 1:** Patient and tumour characteristics

Intrinsic subtype						
	**Luminal A**	**Luminal B**	**Luminal B HER2+**	**HER2+**	**Triple-negative**	**p**

Age group						n.s
Age < 55 year (n = 52)	17 (40)	14 (52)	8 (53)	1 (17)	12 (67)	
Age > 55 year (n = 57)	26 (60)	13 (48)	7 (47)	5 (83)	6 (33)	
Histological grade						< 0.0001
1	16 (37)	2 (8)	1 (7)	0 (0)	0 (0)	
2	26 (61)	6 (22)	6 (40)	3 (50)	3 (17)	
3	1 (2)	19 (70)	8 (53)	3 (50)	15 (83)	
T						n.s.
1	28 (65)	13 (48)	8 (53)	3 (50)	6 (33)	
2	15 (35)	14 (52)	7 (47)	3 (50)	12 (67)	
N						n.s.
0	27 (63)	13 (48)	8 (53)	4 (67)	14 (78)	
1	16 (37)	14 (52)	7 (47)	2 (33)	4 (22)	
% positive cells by immunohistochemistry measured by digital image analysis (mean ± SD)*						
ER	80 ± 13	62 ± 33	52 ± 26	2 ± 1	4 ± 7	< 0.0001
PR	53 ± 31	38 ± 36	19 ± 29	2 ± 2	3 ± 4	< 0.0001
AR	47 ± 20	32 ± 23	28 ± 21	33 ± 15	10 ± 15	< 0.0001
BCL2	56 ± 11	46 ± 24	33 ± 27	7 ± 6	18 ± 15	< 0.0001
HER2	7 ± 11	7 ± 12	37 ± 25	64 ± 19	1 ± 3	< 0.0001
Ki67	14 ± 7	40 ± 17	22 ± 12	31 ± 15	53 ± 16	< 0.0001
p53	13 ± 16	34 ± 32	19 ± 18	17 ± 26	44 ± 35	n.s.
p16	14 ± 8	14 ± 12	14 ± 7	10 ± 4	40 ± 21	< 0.0001
HIF-1α	9 ± 6	12 ± 10	12 ± 10	18 ± 13	18 ± 10	< 0.005
SATB1	12 ± 7	14 ± 10	13 ± 10	10 ± 4	19 ± 18	n.s.

* Statistical significance of variation between the groups tested by one-way ANOVA (logarithm-transformed values of HER2, Ki67, p53, p16, HIF-1α, SATB1 were used for the analysis, however, original values are presented in the table)

**Table 2:** Pairwise correlations between the immunohistochemical markers of ductal carcinoma of the breast

	ER	PR	AR	BCL2	HER2	Ki67	p53	p16	HIF-1α	SATB1
ER	1.0000									

PR	0.5562	1.0000								

AR	0.5651	0.6199	1.0000							

BCL2	0.6950	0.5159	0.4403	1.0000						

HER2	0.0148	-.2411	-.0097	-.1898	1.0000					

Ki67	-.5022	-.3644	-.4497	-.3804	-.1313	1.0000				

p53	-.1546	-.0204	-.0731	-.2154	0.0602	0.1930	1.0000			

p16	-.2583	-.2016	-.1513	-.1058	-.0619	0.1363	0.0264	1.0000		

HIF-1α	-.4606	-.4763	-.5118	-.4665	0.0130	0.3136	0.0245	0.1499	1.0000	

SATB1	-.2651	-.2065	-.3727	-.2572	-.0253	0.0877	0.1345	0.0749	0.5159	1.0000

### Factor analysis of the immunophenotype of the ductal carcinoma of the breast

Factor analysis was performed on 109 patients with a complete set of 10 IHC markers: ER, PR, AR, HER2, BCL2, Ki67, HIF-1α, SATB1, p53, and p16. Rotated factor pattern is presented in the Table 3. Altogether the five factors explained 80% of the variance in the data set.

**Table 3:** Rotated factor pattern of the immunothenotype variation of ductal carcinoma of the breast

	Factor 1	Factor 2	Factor 3	Factor 4	Factor 5
ER	0.83941	-0.01623	0.06273	-0.10947	-0.13607
PR	0.78891	-0.03484	-0.28622	0.17900	-0.09682
AR	0.77439	-0.22905	0.06032	0.10351	0.00616
BCL2	0.76608	-0.06227	-0.21251	-0.21011	0.03420
HER2	-0.08421	-0.06469	0.93842	0.05171	-0.05224
Ki67	-0.70211	-0.23907	-0.38056	0.18840	-0.04474
p53	-0.11614	0.05720	0.03361	0.95603	0.00840
p16	-0.18339	0.01554	-0.04829	0.00668	0.97677
HIF-1α	-0.61104	0.57686	-0.04245	-0.11539	0.02041
SATB1	-0.25600	0.89064	-0.03403	0.11291	0.00695

**Figure 2:**
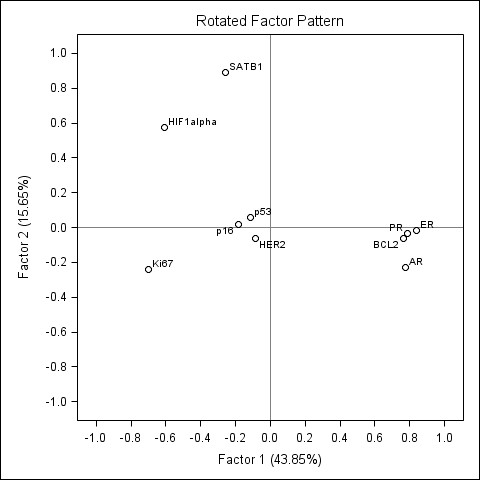
**Rotated factor pattern in the patients with breast ductal carcinoma: loadings of the factors 1 and 2 plotted**. The loadings of the factor 1 (i-Grade) and factor 2 (SATB1/HIF-1α) plotted.

Factors 1 and 2 represented major portion of the variance explained by the five factors extracted (43.9 and 15.7%, respectively). Factor loadings of the factors 1 and 2 are plotted on the Figure 2. The factor 1 is characterized by strong positive loadings of HR (ER, PR, AR) and BCL2 as well as strong negative loadings of Ki67 and HIF-1α. Based on the known biological and prognostic information conveyed by these IHC markers in the context of breast marker and their strong association to the histological grade in our study (see below), this factor pattern can be interpreted as representing a spectrum of "the immunohistochemical grade" (i-Grade): from the tumours with predominant expression of HR and BCL2 (i-Grade-Low) to the tumours with predominant expression of Ki67 and HIF-1α (i-Grade-High).

Factor 2 was characterized by strong positive loadings of SATB1 and HIF-1α (factor loading 0.89 and 0.58, respectively) and was labelled as "SATB1/HIF-1α" (Figure 2).

**Figure 3:**
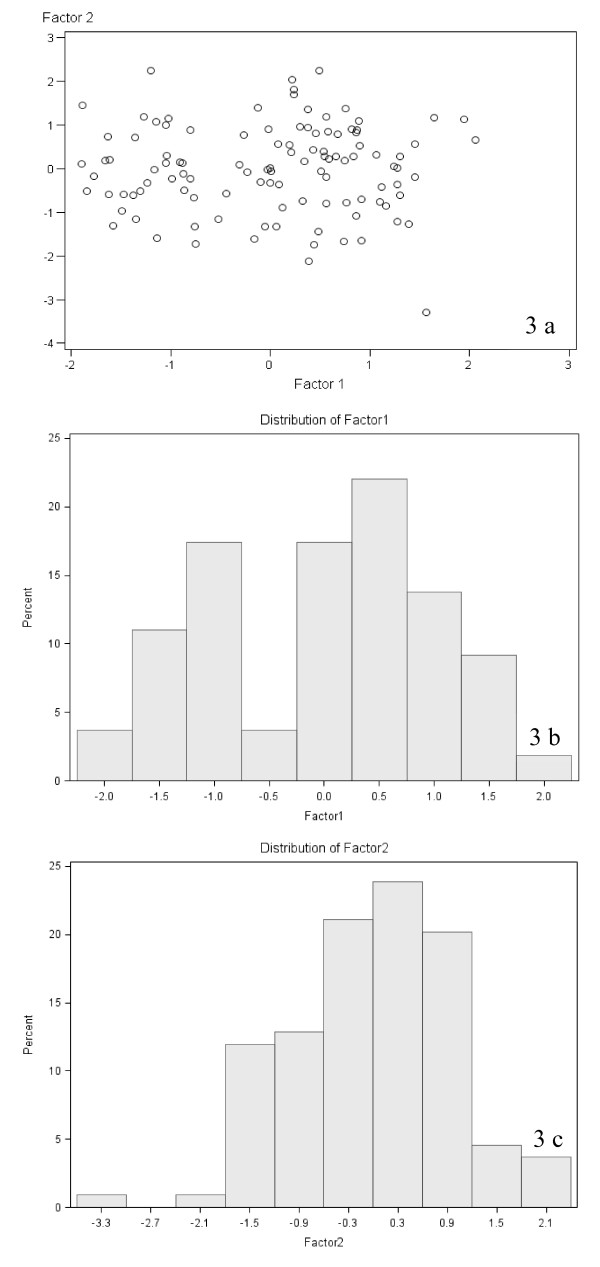
**The distribution of the factor 1 and 2 scores in the patients with breast ductal carcinoma**. **a) **scatter plot of the factor 1 and 2 scores; **b) **histogram of the factor 1 (i-Grade) scores; **c) **histogram of the factor 2 (SATB1/HIF-1α) scores.

The distributions of the factor 1 and 2 scores are plotted (Figure 3a) and represented in the corresponding histograms (Figure 3b and 3c). The histograms reveal bimodal distribution of the factor 1 (i-Grade) and normal distribution of the factor 2 (SATB1/HIF-1α) scores. Accordingly, the heterogeneity of the patient group can be noted in the scatter plot (Figure 3a). To test the associations with clinical and pathologic features, the patients were dichotomized into the i-Grade categories "Low" (factor 1 score > -0.5) and "High" (factor 1 score ≤ -0.5); and the SATB1/HIF-1α expression categories "Low" (factor 2 score ≤ 0) and High (factor 2 score > 0).

Factors 3, 4 and 5 altogether represented the remaining 40.5% (14.6, 13.5, and 12.4%, respectively) of the variance explained by the five factors extracted (Table 3). The factors were characterized by positive loadings of single biomarkers and named accordingly: factor 3 (HER2), factor 4 (p53), and factor 5 (p16). The corresponding factor scores revealed normal distribution (not shown). These factors were dichotomized at the cut-off value of 0.

### Associations between the factor 1 and 2 scores and the conventional categories of the ductal carcinoma of the breast

We explored potential associations between the factor 1 and 2 score categories and the conventional characteristics of the disease: the intrinsic subtype, histological grade (G), tumour stage (T), node status (N), and age group (Table 4).

**Table 4:** Associations between the factor 1 and 2 scores and the conventional categories of the ductal carcinoma of the breast

	n	i-Grade-High	SATB1/HIF-1α-High	HER2-High	p53-High	p16-High
Number of patients 109						
Age group		n.s.	n.s.	*p <*0.03	*p <*0.003	n.s.
Age < 55 year	52	19 (37)	28 (54)	20 (38)	39 (75)	28 (54)
Age > 55 year	57	17 (30)	29 (51)	34 (60)	27 (47)	30 (53)
Histological grade		*p <*0.00001	n.s.	n.s.	n.s.	n.s.
1	19	0 (0)	12 (63)	10 (53)	7 (37)	12 (63)
2	44	9 (21)	26 (59)	27 (61)	30 (68)	22 (50)
3	46	27 (59)	19 (41)	17 (37)	29 (63)	24 (52)
T		n.s.	n.s.	n.s.	n.s.	n.s.
1	58	16 (28)	31 (53)	33 (57)	37 (64)	27 (47)
2	51	20 (44)	26 (51)	21 (41)	29 (57)	31 (61)
N		n.s.	n.s.	n.s.	n.s.	n.s.
0	66	25 (38)	36 (55)	30 (45)	40 (61)	35 (53)
1	43	11 (26)	21 (49)	24 (56)	26 (60)	23 (53)
Intrinsic subtype		*p <*0.0001	n.s.	*p <*0.00001	n.s.	*p <*0.002
Luminal A	43	0 (0)	29 (67)	25 (58)	23 (53)	26 (60)
Luminal B	27	7 (26)	10 (37)	10 (37)	19 (70)	8 (30)
Luminal B HER2+	15	6 (40)	7 (47)	12 (80)	10 (67)	8 (53)
HER2+	6	6 (100)	2 (33)	6 (100)	2 (33)	1 (17)
Triple-negative	18	17 (94)	9 (50)	1 (6)	12 (67)	15 (83)

The factor 1 (i-Grade) was associated with the intrinsic subtypes (*p <*0.0001): all cases of HER2-positive (n = 6) and all but one (94%) Triple-negative carcinoma fell into the i-Grade-High category. Significant proportion of Luminal B (26%) and Luminal B HER2 positive (40%) cases but none of Luminal A type (n = 43) were i-Grade-High.

**Figure 4:**
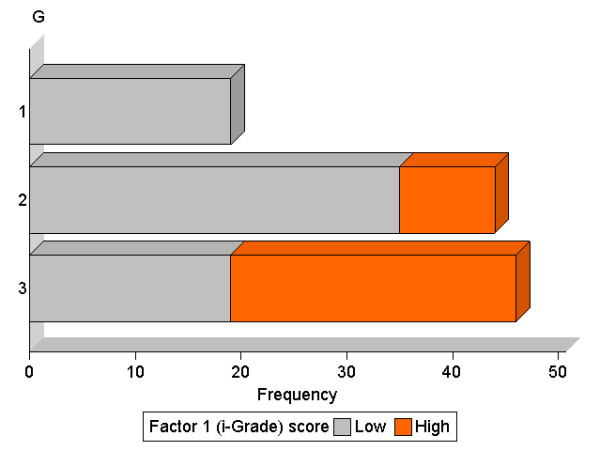
**Association between the i-Grade and the histological grade (G)**. The bar chart represents the distribution of i-Grade-Low (grey) and i-Grade-High (orange) tumours against the histological grade (G1, 2, and 3)

Association between the i-Grade and the G was highly significant (*p <*0.0001, Figure 4): all cases of G1 were i-Grade-Low (n = 19) whereas G2 and G3 were increasingly i-Grade-High (21%, and 58%, respectively). The i-Grade was not significantly associated with T, N, or patient age group.

Factor 3 (HER2) was associated with older age and presented relevant associations with the intrinsic subtypes (Table 4), however, significant proportion of Luminal A and B and one Triple-negative case were in the HER2-High category. This is related to the relatively low HER2-High cut-off value (0) used for dichotomization of the factor score.

Factor 5 (p16) was remarkable presenting with high values in the majority (83%) of TN cases. Factor 2 (SATB1/HIF-1α) and factor 4 (p53) were not significantly associated with any of the categories tested (Table 4).

**Figure 5:**
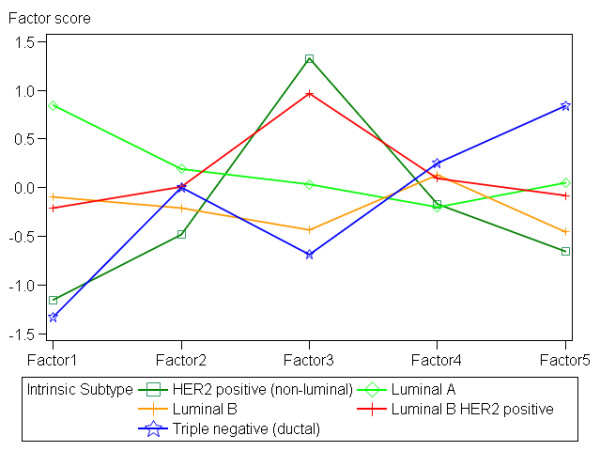
**Representation of the factor score profiles of the intrinsic subtypes**. The multiple line chart outlines the factor score profiles of the intrinsic subtypes. The lines connect the mean values of the factor 1-5 scores obtained by one-way ANOVA for each intrinsic subtype as explanatory variable.

The IHC profile of the intrinsic subtypes was further highlighted by one-way ANOVA with the factor scores used as dependent variables. Summary of the profiles is plotted on Figure 5. Significant (*p <*0.0001) ANOVA models were obtained for the factors 1, 3, and 5. Remarkably, the (1) Luminal A, (2) Luminal B with Luminal B HER+, and (3) HER2+ with the Triple-negative tumours formed three distinct subgroups of the factor 1 distribution (relevant pairwise comparisons of the three groups were statistically significant). Factor 3 (HER2) distribution was concordant with the definition of the intrinsic subtypes; although Luminal A showed slightly higher values than Luminal B, the pairwise comparison did not reveal significant difference. Factor 5 (p16) was remarkable for high values in the TN tumours, significantly (*p <*0.05) higher than in all other intrinsic subtypes. Pairwise comparisons of the p16 variance presented the same significant differences (Table 1).

### Factor analysis of the immunophenotype of the HR-positive ductal carcinoma of the breast

**Figure 6:**
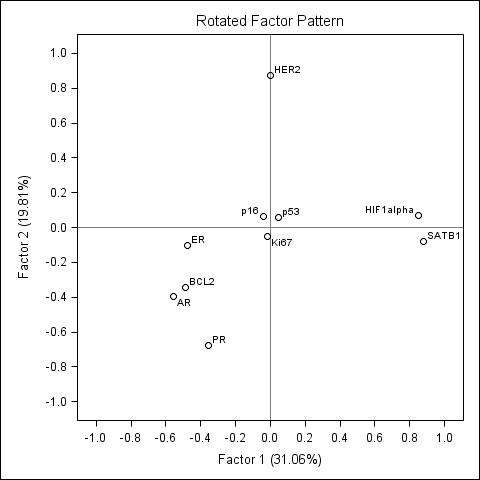
**Rotated factor pattern in the patients with hormone receptor positive breast ductal carcinoma: loadings of the factors 1 and 2 plotted**. The loadings of the factor 1 (SATB1/HIF-1α - AR/ER/BCL2) and factor 2 (HER2-PR) plotted.

**Figure 7:**
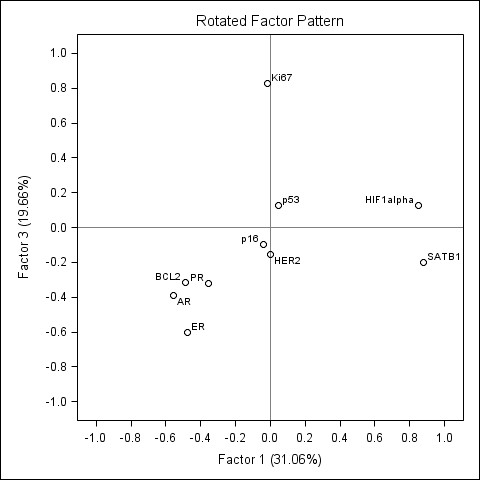
**Rotated factor pattern in the patients with hormone receptor positive breast ductal carcinoma: loadings of the factors 1 and 3 plotted**. The loadings of the factor 1 (SATB1/HIF-1α - AR/ER/BCL2) and factor 2 (Ki67-ER) plotted.

To explore the immunophenotype interactions in the group of HR-positive tumours, we performed factor analysis in 85 cases of Luminal A, Luminal B, and Luminal B HER2 positive tumours (the analysis of 70 patients with Luminal A and B only gave similar results and is not presented here). The rotated factor pattern is presented in the Table 5, Factor 1 and 2 loadings are plotted on Figures 6 and 7. Altogether the five factors explained 77% of the variance in the data set.

**Table 5:** Rotated factor pattern of the immunothenotype of the hormone receptor positive ductal carcinoma of the breast

	Factor 1	Factor 2	Factor 3	Factor 4	Factor 5
ER	-0.47598	-0.10090	-0.60245	-0.10337	-0.16151
PER	-0.35541	-0.67714	-0.31955	0.29194	0.00229
AR	-0.55499	-0.39729	-0.39127	0.24653	0.27274
BCL2	-0.48636	-0.34242	-0.31599	-0.38459	-0.01865
HER2	0.00251	0.87192	-0.15417	0.25187	0.08731
Ki67	-0.01734	-0.04969	0.82934	0.10271	-0.15723
p53	0.04843	0.05677	0.12929	0.88141	-0.12610
p16	-0.03786	0.06404	-0.09880	-0.12972	0.95100
HIF-1α	0.84950	0.06950	0.12918	-0.09585	0.05170
SATB1	0.87715	-0.08281	-0.20321	0.16228	-0.07999

The factor 1 (31.1% of variation) resembled the factor 2 (SATB1/HIF-1α) in the whole group of the patients, however, the factor pattern was different: it was characterized by strong positive loading of HIF-1α, identical to that of SATB1, and by moderate negative loadings of AR, ER, and BCL2. This pattern suggests inverse relation between SATB1 and HIF-1α co-expression and the co-expression of AR, ER, and BCL2 in the subgroup of HR-positive tumours. Since the biological meaning of this interrelation is not clear, this factor is labelled "SATB1/HIF-1α-AR/ER/BCL2".

The factors 2 and 3 contributed a similar proportion of variation (19.8 and 19.7%, respectively) to the data set. Factor 2 was characterized by positive HER2 (0.87) and negative PR (-0.68) loadings, while factor 3 - by positive Ki67 (0.83) and negative ER (-0.60) loadings. Respectively, these factors were labelled "HER2-PR" and "Ki67-ER".

Factors 4 (p53) and 5 (p16) resembled those already identified in the whole group of patients and contributed 15.7 and 13.8% of variation, respectively.

All factor scores revealed a normal distribution (not shown) and were dichotomized at the cut-off value of 0.

### Associations between the factor scores and the conventional categories of the HR-positive ductal carcinoma of the breast

Factor 1 (SATB1/HIF-1α-AR/ER/BCL2) score categories were not associated with the patient's age group, T, N, G, or intrinsic subtype (Table 6). Factor 2 (HER2-PR) score High category was associated with the older patients' age group (*p <*0.002) and intrinsic subtype (*p <*0.05).

**Table 6:** Associations between the factor scores and the conventional categories of the hormone receptor positive ductal carcinoma of the breast

	n	i-IHC-High	SATB1-High	HER2-High	p53-High	p16-High
Number of patients	85					
Age group		n.s.	*p <*0.0015	n.s.	*p <*0.003	n.s.
Age < 55 year	39	12 (31)	12 (31)	22 (56)	30 (77)	20 (51)
Age > 55 year	46	12 (26)	30 (65)	18 (39)	21 (46)	25 (54)
Histological grade		n.s.	*p <*0.2951	*p <*0.0001	n.s.	n.s.
1	19	6 (32)	7 (37)	3 (16)	8 (42)	9 (47)
2	38	10 (26)	22 (58)	14 (37)	25 (66)	22 (58)
3	28	8 (29)	13 (46)	23 (82)	18 (64)	14 (50)
T		n.s.	n.s.	n.s.	n.s.	n.s.
1	49	13 (27)	28 (57)	22 (45)	32 (65)	23 (47)
2	36	11(31)	14 (39)	18 (50)	19 (53)	22 (61)
N		n.s.	n.s.	n.s.	n.s.	n.s.
0	48	14 (29)	23 (48)	23 (48)	28 (58)	25 (52)
1	37	10(27)	19 (51)	17 (46)	23 (62)	20 (54)
Intrinsic subtype		n.s.	*p <*0.0331	*p <*0.0001	n.s.	n.s.
Luminal A	43	13 (30)	19 (44)	7 (16)	21 (49)	22 (51)
Luminal B	27	7(26)	11 (41)	23 (85)	19 (70)	12 (44)
Luminal B HER2+	15	4 (27)	12 (80)	10 (67)	11 (73)	11 (73)

**Figure 8:**
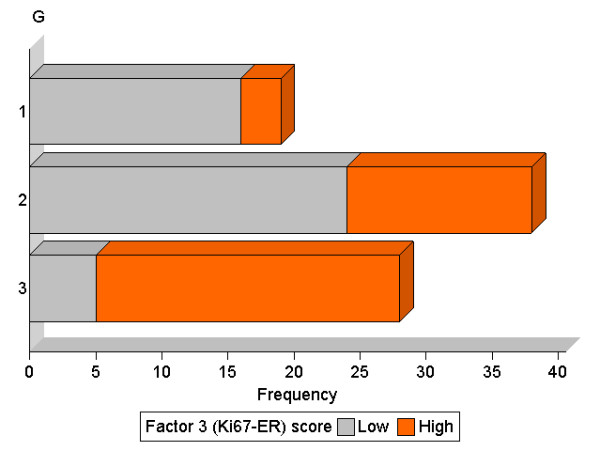
**Association between the Ki67-ER and the histological grade (G)**. The bar chart represents the distribution of Ki67-ER-Low (grey) and Ki67-ER-High (orange) tumours against the histological grade (G1, 2, and 3).

Associations of the factor 3 (Ki67-ER) score categories closely resembled those of the factor 1 (i-Grade) in the whole group of patients: high scores were increasing with the histological grade (*p <*0.0001, Figure 8) and more frequently found in Luminal B subtypes (*p <*0.0001). Remarkably, some Ki67-ER-High tumours were detected in both G1 (3/19, 16%) and Luminal A (9/43, 21%) categories.

## Discussion

Our study proves that important biological interdependencies can be detected at the level of tumour tissue immunophenotype based on the multivariate analysis of DA data. In a cohort of 109 patients with ductal carcinoma of the breast, we were able to detect biologically relevant interdependencies and heterogeneity largely reflecting the main intrinsic subtypes of the disease and providing new data and insights into the breast cancer biology.

The design of our study enabled us to avoid significant human impacts and assumptions while obtaining the results: we performed an automated image analysis with automated detection of tumour tissue of the ductal carcinoma of the breast TMAs stained for 10 IHC markers, followed by factor analysis of the data set. In some sense, our results represent an automated readout of the IHC data in the TMAs. Factor analysis revealed latent factors governing the interdependent variance of the immunophenotype: being orthogonally independent by definition, the factors can be seen as independent biological processes standing behind the IHC profile variability in the disease entities. We then produced integral characteristics (factor scores) for individual patients and tested their associations with main conventional categories of the breast ductal carcinoma.

The factors of the immunophenotype variance, established in our study, are in line with the current knowledge of breast cancer biology, however, new insights emerge.

We found that major factor of the IHC profile variation in the ductal breast carcinoma was characterized by a strong inverse relation between the expression of hormone receptors (ER, PR, AR) along with anti-apoptotic marker BCL2, on one side, and Ki67 (proliferation) and HIF-1α (hypoxic stress, angiogenesis, see below), on the other side. While the corresponding correlations were detected by the pairwise correlation analysis (Table 2), they were only moderate, true interdependencies being obscured by multiple interactions in the dataset and difficult to interpret. We named the factor 1 the "i-Grade" since its pattern reflected the interdependent variance of the IHC markers known to represent the axis from aggressive (Ki67, HIF-1α) to more indolent (HR, BCL2) behaviour of the disease [10]. In particular, biological meaning of this factor includes the axis of anti-apoptotic ← → proliferative effects that could be indicative of the variance of the tumour growth behaviour. Importantly, the anti-apoptotic effects were closely related to the expression of HR, while proliferative effects were paralleled by the marker of increased hypoxic stress and angiogenesis (HIF-1α). Also, our interpretation of the biological nature of the factor 1 (i-Grade) was further confirmed by pronounced bimodal distribution of the factor scores and strong associations with higher histological grade and the more aggressive intrinsic subtypes (HER2 positive and Triple-negative).

The pattern of the factor 1 (i-Grade) reveals important interactions between the HR, BCL2, Ki67, and HIF-1α. Our data support the notion that BCL2 may be a useful addition to the current scoring schemes as reported recently by Dawson et al. [19]. Furthermore, a combined mitotic/BCL2 or Ki67/BCL2 index reflects true biological variation in breast cancer and may provide more relevant prognostic information [14,20]. These latter observations were based on a combination of semi-quantitative scores of the two biomarkers; Ki67/BCL2 index was represented by subtraction of Ki67-BCL2 scores. As a matter of fact, factor 1 (i-Grade) scores showed very strong correlation (r = 0.89, *p <*0.0001, data not shown) with the difference between Ki67 and BCL2 expression (the percentage of Ki67 positive cells - the percentage of BCL2 positive cells) in our data set. We therefore provide quantitative and multivariate analysis-based evidence supporting the suggested semi-quantitative and empirical definition of the Ki67/BCL2 index [14]. In addition, our data suggest that inclusion of HIF-1α into this integrated index of the disease aggressiveness might bring more accuracy to this potential prognostic indicator.

Factor analysis, performed in the HR-positive subgroup of cases (LA, LB, and LB HER2-positive), extracted factor 3 (Ki67-ER) resembling the i-Grade by its associations to the histological grade and the intrinsic subtypes. The peculiarities of this "aggressiveness index" in the HR-positive subpopulation of breast cancer should be noted. First, the factor 3 (Ki67-ER) was no longer the main source of variability and did not present with bimodal distribution of the score values. This change can be explained by the decreased variation of Ki67 expression in the data set after exclusion of the most proliferative subtypes (Triple-negative and HER2 positive). Second, the pattern of the factor 3 suggests that Ki67/ER index rather than Ki67/BCL2 index might be a more accurate measure of the aggressiveness of the HR-positive disease. Or, at least, ER is tightly co-expressed with BCL2 and therefore sufficient to be used in combination with Ki67 in the HR-positive tumours. Although clinical utility of BCL2 as an independent prognostic marker has been suggested [19,21,22], this notion has to undergo scrutiny of large prospective trials and multivariate analyses. Third, although the Ki67-ER was strongly associated with the histological grade and intrinsic subtypes, it did attribute to the Ki67-ER-High category 16 and 21% cases of the Grade 1 and Luminal A tumours, respectively. This suggests that the combined index may provide an added value to the conventional categories securing against potential misinterpretations in, at least, borderline cases. It has been reported that Ki67 index can classify G2 breast cancer into low and high risk subgroups [23]. It has been shown that image analysis of Ki67 correlates strongly with human evaluation, however, evaluation bias is possible and the results are potentially dependent on different Ki67 antibodies used [24]. Therefore, improved stratification based on combinatorial index could be useful for better discrimination of Luminal A and B subtypes, in particular, and decision on chemotherapy. Interestingly, suggestions to develop specific sets of prognostic IHC biomarkers in lymph node-negative and positive breast cancer subgroups [25] also can be viewed as combinatorial approach, leading ultimately to the concept of multimodal metamarkers of the disease [13].

HER2 expression was independent of other IHC parameters in the patients with ductal carcinoma, however, in the HR-positive subset, HER2 "competed" mostly with PR but not ER expression (Figure 6). This finding is intriguing since the independent significance of ER and PR (as well as BCL2) is not entirely clear because of significant correlations between these biomarkers. Loss of PR expression might indicate somewhat worse prognosis compared to ER+/PR + tumours, whereas various combinations of BCL2, p53, HER2 expression might provide additional prognostic information as recently reviewed by Rakha et al. [10]. In our study, we have found relevant pairwise correlations between ER, PR, and BCL2, however, factor analysis sheds the light into their true interdependencies: in the context of ductal breast carcinoma, we confirm that the HR and BCL2 expression is indeed highly inter-dependent and governed by the same latent factor of variation which is also characterized by inverse relation to Ki67 and HIF-1α. In the context of HR-positive tumours, high ER expression is seen in less proliferative (Ki67) cases, whereas low PR expression may indicate higher HER2 expression. Therefore, differential ER and PR expression may reflect differences in proliferative activity and HER2 expression of the HR-positive tumours; consequently, carrying related prognostic information (if the continuous increase of HER2 expression could be viewed as a potential feature of more aggressive behaviour). As noted already\, our analyses suggest that BCL2 correlates closely with the expression of hormone receptors (both in the whole group of patients and HR-positive tumours) and does not carry an independent information in the data sets.

Our study highlights the potential significance of relatively new and less-explored biomarkers in breast cancer. SATB1, a genome organizer that recruits chromatin-remodelling enzymes to regulate chromatin structure and gene expression, has been recently implicated to promote growth and metastasis of breast cancer and indicate poor prognosis [26,27]. It was not confirmed by other studies and remains controversial as a prognostic factor and a potential target for therapy [28-31]. In particular, the expression levels of SATB1 mRNA in 2058 breast cancer samples were not related to disease-free survival among ER negative cancers, however, high SATB1 expression among ER positive tumours showed beneficial prognosis; nevertheless, even in ER positive cancer no independent prognostic value in multivariate analysis with standard parameters was observed [31]. In the study of Patani et al., high SATB1 expression levels were more often found in ER negative tumour samples [27]. Our study presents first evidence on correlation of IHC expression of SATB1 with ER and other markers. The factor 1 (SATB1/HIF-1α-AR/ER/BCL2) pattern in the HR-positive tumours revealed an inverse relation between the two groups of markers with the opposite factor loadings. Indeed, it is likely that the prognostic effects of SATB1 can be caused by a possible confounding effect of its inverse relation to ER expression [31]. Furthermore, we did not find any associations of SATB1 expression with the histological grade or other categories tested. Nevertheless, it is remarkable that SATB1 was closely associated with HIF-1α in HR-positive and the whole cohort of ductal carcinoma; the factors with involvement of SATB1 and HIF-1α caused a major portion of variation in both groups, especially, in HR-positive tumours. This implies that SATB1 and HIF-1α may be important markers of the disease, whereas their biological and clinical significance remains to be elucidated.

HIF-1α is broadly expressed in many human cancers and frequently correlates with poor prognosis; it affects many key aspects of tumour initiation, progression, invasion, inflammatory cell recruitment and metastasis, and represents an attractive target for anti-cancer therapies as reviewed recently [32]. Yamamoto et al. [33] reported on 171 cases of invasive breast cancer examined, nuclear HIF-1α expression was detected in 37% cases (a cut-off of 5% was used as in previous study [34]). HIF-1α was closely associated with indicators of aggressive phenotype, such as high histological grade, lymph node metastasis, large tumour size, high proliferation rate, negativity of hormone receptors, HER2 positivity and increased VEGF expression; elevated levels of HIF-1α expression were associated independently with shorter disease-free and overall survival [33]; hypoxia and HIF-1α might be related to the worse prognosis found in CD44 + CD24-/low positive breast tumors [35]. Association of HIF-1α expression with unfavourable prognosis in patients with breast cancer has been demonstrated by previous studies [34,36,37]. In our study, we confirm the association of HIF-1α and Ki67 expression, both by pairwise correlation and similar factor loadings on the factor 1 (i-Grade) in the group of ductal carcinoma. Also, the i-Grade scores were strongly associated with histological grade, therefore, confirming aggressive nature of HIF-1α and Ki67 (confirmed also by one-way ANOVA using HIF-1α and Ki67 as dependent variables, data not shown). However, interpretation of the factor patterns in our analyses presents some peculiarities. First, in the group of ductal carcinoma, we find that HIF-1α participates in two factors: factor 1 (i-Grade) along with Ki67 and factor 2 - along with SATB1. In the group of ductal carcinoma, we find that HIF-1α participates in two factors: factor 1 (i-Grade) along with Ki67 and factor 2 - along with SATB1. Second, in the group of HR-positive ductal carcinoma, HIF-1α participates in the most important factor 1 along with SATB1 providing factor loadings opposite to those of AR, ER and BCL2, independently of Ki67 expression (factor 3). This further supports the notion that HIF-1α and SATB1 may convey important biological messages other than the aggressiveness of the disease reflected by Ki67 expression and histological grade, at least in HR-positive disease.

Expression of p16 was governed by an independent factor in our analyses and was remarkable for significantly higher levels in the TN subtype compared to all other intrinsic subtypes. We therefore support the reports [38-41] on increased p16 expression in basal/triple-negative breast cancer also suggesting frequent inactivation of the retinoblastoma tumour suppressor (Rb) and up regulation of the cyclin-dependent kinase inhibitor p16 in these tumours. Furthermore, down regulation of p16 expression has been observed in some basal-like breast cancer cell lines, suggesting that such cells can be divided into two groups according to Rb and p16 status, predictive of reduced chemo sensitivity in p16 depleted cancers [42]. Interestingly, factor analysis performed in the subgroup of 17 patients with Triple-negative tumours (data not shown), revealed strong association of p16 expression with Ki67 and their strong inverse relation to AR (but not ER or PR) expression. AR expression has been reported as a marker of better prognosis in Triple-negative breast cancer [43-45], however, our findings warn that this effect may be caused by the confounding effect due to the inverse relation between AR and Ki67 with p16. This data awaits confirmation on a larger set of patients.

In our study we extracted 5 factors from the dataset of 10 IHC markers, arriving to clinically and biologically meaningful interpretation of the results. However, since the results of factor analysis may be influenced the number of factors extracted, defined by the investigator, we have also tested the robustness of our results by extracting 3 or 4 factors (not shown). The pattern of 4 factors extracted was largely the same, except p16 loadings moderately contributing to the i-Grade (-0.41) or Ki67-ER (0.49), in all or HR-positive tumours respectively. Similarly, extraction of 3 factors resulted in redistribution of p53 loadings whereas the interactions of the other markers remained essentially stable.

In summary, our study demonstrates that factor analysis of multiple IHC biomarkers measured by automated DA is an efficient exploratory tool clarifying complex interdependencies in the breast ductal carcinoma IHC profiles. We find that a major factor of the aggressive disease behaviour (i-Grade) is characterized by opposite loadings of ER/PR/AR/BCL2 and Ki67/HIF-1α. The i-Grade factor scores represent integral quantitative characteristics that reveal bimodal distribution and are strongly associated with the histological grade and relevant intrinsic subtypes. In HR-positive tumours, the aggressiveness of the tumour is best reflected by the combination of Ki67 and ER, rather than Ki67 and BCL2. These indices may provide additional risk stratifications for the currently used grading systems whereas their clinical utility and cut-offs remain to be established by analysis of clinical outcomes. Interestingly, we found inverse relation between HER2 and PR expression in the HR-positive tumours which, along with the inverse relation between Ki67 and ER, may shed the light into the differential information conveyed by the ER and PR expression. Remarkably, SATB1 along with HIF-1α reflected the second major factor of variation in our patients; furthermore, in the HR-positive group they were inversely associated with the HR and BCL2 expression and represented the major factor contributing to the variation in the IHC data set. However, this factor was not associated with the clinicopathologic categories studied. Biological meaning of this variation remains unclear: HIF-1α and SATB1 may convey important biological messages other than the aggressiveness of the disease reflected by Ki67 expression and histological grade. Meanwhile, we support the notion that the suggested prognostic significance of SATB1 may be related to its inverse relation to the ER expression. Finally, our analysis confirms high expression levels of p16 in Triple-negative tumours.

## Abbreviations

AR: The androgen receptor; DA: Digital image analysis; DCIS: Ductal carcinoma in situ; ER: The estrogen receptors; FDA: Food and Drug Administration; FISH: Fluorescence in situ hybridization; G: Histological grade; HER2: The human epidermal growth factor receptor 2; HIF1α: The hypoxia-inducible factor 1α; HR: The hormone receptors; i-Grade: The "immunohistochemical grade"; IHC: Immunohistochemistry; PR: The progesteron receptors; N: Lymph node involvement; SATB1: Special AT-rich sequence-binding protein 1; T: Tumour stage; TMA/TMAs: Tissue microarray/Tissue microarrays.

## Competing interests

The authors declare that they have no competing interests.

## Authors' contributions

ArL and AiL contributed equally to the study. ArL drafted the manuscript, performed statistical analysis. AiL designed and carried out the TMAs digital analyses and edited the manuscript. DD performed visual evaluation of the TMAs images. All authors participated in conception and design of the study, reviewing the analysis results, read and approved the final manuscript.
